# Rutinosides-derived from *Sarocladium strictum* 6-*O*-α-rhamnosyl-β-glucosidase show enhanced anti-tumoral activity in pancreatic cancer cells

**DOI:** 10.1186/s12934-024-02395-0

**Published:** 2024-05-08

**Authors:** Gisela Weiz, Alina L. González, Iara S. Mansilla, Martín E. Fernandez-Zapico, María I. Molejón, Javier D. Breccia

**Affiliations:** 1 Facultad de Ciencias Exactas y Naturales, Instituto de Ciencias de la Tierra y Ambientales de La Pampa (INCITAP), Universidad Nacional de La Pampa-Consejo Nacional de Investigaciones Científicas y Técnicas (UNLPam-CONICET), Av. Uruguay 151, 6300 Santa Rosa, La Pampa Argentina; 2 Schulze Center for Novel Therapeutics, Division of Oncology Research, Mayo Clinic, Rochester, MN 55905 USA

**Keywords:** Diglycosidases, Glycoconjugates, Transglycosylation, Therapeutics, Pancreatic cancer

## Abstract

**Background:**

Low targeting efficacy and high toxicity continue to be challenges in Oncology. A promising strategy is the glycosylation of chemotherapeutic agents to improve their pharmacodynamics and anti-tumoral activity. Herein, we provide evidence of a novel approach using diglycosidases from fungi of the *Hypocreales* order to obtain novel rutinose-conjugates therapeutic agents with enhanced anti-tumoral capacity.

**Results:**

Screening for diglycosidase activity in twenty-eight strains of the genetically related genera *Acremonium* and *Sarocladium* identified 6-*O*-α-rhamnosyl-β-glucosidase (αRβG) of *Sarocladium strictum* DMic 093557 as candidate enzyme for our studies. Biochemically characterization shows that αRβG has the ability to transglycosylate bulky OH-acceptors, including bioactive compounds. Interestingly, rutinoside-derivatives of phloroglucinol (PR) resorcinol (RR) and 4-methylumbelliferone (4MUR) displayed higher growth inhibitory activity on pancreatic cancer cells than the respective aglycones without significant affecting normal pancreatic epithelial cells. PR exhibited the highest efficacy with an IC_50_ of 0.89 mM, followed by RR with an IC_50_ of 1.67 mM, and 4MUR with an IC_50_ of 2.4 mM, whereas the respective aglycones displayed higher IC_50_ values: 4.69 mM for phloroglucinol, 5.90 mM for resorcinol, and 4.8 mM for 4-methylumbelliferone. Further, glycoconjugates significantly sensitized pancreatic cancer cells to the standard of care chemotherapy agent gemcitabine.

**Conclusions:**

αRβG from *S. strictum* transglycosylate-based approach to synthesize rutinosides represents a suitable option to enhance the anti-proliferative effect of bioactive compounds. This finding opens up new possibilities for developing more effective therapies for pancreatic cancer and other solid malignancies.

**Supplementary Information:**

The online version contains supplementary material available at 10.1186/s12934-024-02395-0.

## Background

Despite significant advances in the field of developmental therapeutics leading to many of FDA approved regimens in Oncology, these approaches are far from optimal due to high systemic toxicity, and modest targeting efficacy. This is in part due to low tumor selectivity and pharmacokinetic drawbacks, including instability of the molecules and poor water solubility [1–3]. Glycosylation represents a promising strategy to improve cellular and tissue targeting selectivity, efficacy, and stability of anti-tumoral agents [4–9].

The approaches to chemically synthesize complex-sugars glycoconjugates are well established, however, chemical derivatization suffers from major difficulties in achieving regio- and stereoselectivity due to labor-intensive activation and protection procedures, multistep synthetic routes with low overall yields and the use of toxic catalysts and solvents [10–13]. To overcome these limitations, enzymatic glycosylations have emerged as an alternative strategy since enzymes are inherently selective providing efficient approaches to modify small molecules via covalent attachment of glycosyl residues [14–17]. The major enzymes catalyzing the coupling of carbohydrates are glycoside hydrolases (GHs) and glycosyltransferases (GTs) [18–20]. Although highly efficient, GTs have several drawbacks associated with the high costs, low stability, and difficult or limited availability of nucleotide sugar donors. By contrast, GHs are extremely common, versatile, stable, can be derived from a number of sources, and have wide range of substrate specificities.

Among the GHs (EC 3.2.1.x), diglycosidases with synthetic activities [21–23], represent a suitable class of enzyme to generate glycoconjugates aimed at improving the efficacy of known anti-cancer agents. These enzymes are found in microorganisms and plants; fungal extracellular diglycosidases are among the most commonly used to perform glycosylation reactions with relatively high yields [24–27]. Herein, we identified and biochemically characterized a diglycosidase from twenty-eight strains of the genera *Acremonium* and *Sarocladium* [28]. This enzyme has capacity to transglycosylate bulky OH-acceptors, including bioactive compounds. A comparison of the glycoconjugates with its aglycones revealed increased anti-cancer activity over the parental drug in pancreatic cancer cells and to a lesser extent on liver cancer models. The synthetic simplicity of diglycosidases suggest that enzymatic glycosylation of anti-tumoral agents might form the basis of a novel strategy for the development of cancer-targeted therapies.

## Materials and methods

### Chemicals

4-methylumbelliferone, hesperidin, rutin, hesperidin methylchalcone, isoquercetin, resorcinol, phloroglucinol, methanol, ethanol, butanol, *n*-propanol, isopropanol, isoamyl alcohol, 2-phenylethanol, *p*-Nitrophenyl-β-d-glucopyranoside and *p*-Nitrophenyl-α-l-rhamnopyranoside were purchased in Sigma Chemical (Sigma, St. Louis, MO, USA). Narcissin, tulipanin and myrtillin were purchased in Extrasynthese company (Extrasynthese, Genay Cedex, France). HPLC-grade methanol (LiChrosolv^®^) was obtained from Merck (Merck, Darmstadt, Germany). The 4-methylumbelliferyl-rutinoside reagent was synthesized as described in Mazzaferro et al. [29].

### Cultivation of *Acremonium* and *Sarocladium* strains and diglycosidase activity screening

Twenty-eight *Acremonium* and *Sarocladium* strains were kindly provided from Dr. Alejandra Hevia, (Micology Department, INEI ANLIS Dr. Carlos G. Malbrán, Buenos Aires, Argentina). The strains were cultured in media containing (g L^−1^): 5.0 flavonoid (hesperidin, rutin or diosmin), 1.0 milk peptone, 2.0 yeast extract, 50 mM sodium citrate buffer pH 5.0 and 15.0 agar. Carbon source assessment was estimated from the radial rates of colony growth (*K*r) and the clarification halo around colonies (*K*c) during growth for 20 days at 25 °C. Mycelial growth and clarification halos were analyzed using the software ImageJ (National Institutes of Health, USA). 

To determinate diglycosidase activity, pieces of agar from the clarification zone around the colonies cultivated in the selection medium were used as the source of enzymatic activities. The agar samples were processed according to González et al. [30]. For zymographic analysis, native PAGE electrophoresis was performed according to Laemmli [31] and developed with the fluorogenic substrate 4-methylumbelliferyl-rutinoside previously synthetized in our group [29].

### Production and purification of 6-*O*-α-rhamnosyl-β-glucosidase

Submerged cultures of *S. strictum* DMic 093557 were grown in a 5 L bioreactor (Braun, Stuttgart, Germany) at an agitation speed of 250 rev min^−1^ and an aeration of 0.4 vvm at 25 °C using the following culture medium (g L^−1^): 2.5 rutin; 1.0 milk peptone, 2.0 yeast extract and 30 mM sodium citrate buffer. The pH was maintained at 5.0 by automated addition of 0.5 M H_2_SO_4_ or 1 M NaOH. The bioreactor was inoculated with 10 mL of a 24 h culture grown in Luria–Bertani (LB) medium. The culture broth was filtered through Whatman N^o^. 1 filter paper. It was then precipitated with ammonium sulfate to 56.5% saturation and centrifuged (20 min, 20,000 rpm at 4 °C). After resuspension in 5 mM sodium citrate pH 5.0, the precipitate was subjected to gel filtration chromatography (Sephadex G-75); elution was performed using an isocratic flow with the same buffer. Active fractions were pooled, lyophilized and stored at − 18 °C. To estimate the location of the enzyme, the fungal mycelium after nine days of growing was centrifuged (10 min, 12,000×*g*, 25 °C). Pellets were washed twice with 0.9% w v^−1^ NaCl and resuspended in 5 mM sodium citrate buffer (pH 5.0). Percentage of 57.48 ± 4.22% belonged to the extracellular space and 55.17 ± 5.70% corresponded to cell-attached enzymatic activity. Since the stability of the enzyme, the culture supernatant was selected to purification.

### SDS-PAGE and protein sequencing

The proteins were visualized by Coomassie Brilliant Blue G-250 staining (BioRad, Hercules, Calif. 1610406) and molecular mass was estimated (BioRad, Hercules, Calif. 161-0373) [35]. Individual bands on the SDS-PAGE were excised from the gel and subjected to mass spectrometry analyses carried out by CEQUIBIEM (Buenos Aires University, Buenos Aires, Argentina). Protein identity from peptide mass fingerprints was determined by the MASCOT program (Matrix Science Inc. http://www.matrixsciende.com/search-form-select.html) Fragmentation was carried out with more intense MS peaks (MS/MS). The molecular weight and isoelectric point were performed using ExPASy [36] and the secretory signal sequences of αRβG were predicted using SignalP [37]. Protein content was determined in all cases by the bicinchoninic acid method using bovine serum albumin (BSA) as standard for the calibration curves [38]. All determinations were done in duplicate.

### Enzyme assays and kinetic parameters

For monitoring of the hydrolysis reaction, 20 μL of suitably diluted enzyme was added to 230 μL of 0.11% (w v^−1^) of substrate in 50 mM sodium phosphate buffer pH 6.0, and the reaction mixture was incubated at 60 °C for 1 h. The total amount of reducing sugars produced during enzymatic deglycosylation of the substrates was determined using 2,4-dinitrosalicylic acid [32]. Since the substrates rutin and isoquercetin interfered with the reducing sugar methods, the enzymatic activity was measured according to Weiz et al. [33]. One unit of αRβG activity was defined as the amount of enzyme required to release 1 μmol of product per min at 60 °C. The effect of pH and temperature on the enzyme activity was assessed with rutin as a substrate. For reaction at different pH values, the following buffers were used (50 mM): sodium citrate (pH 4.0–5.0), sodium phosphate (pH 6.0–8.0) and Tris–HCl (pH 9.0). Then the reaction was performed at different temperatures (4–70 °C) using a 50 mM sodium phosphate buffer, pH 6.0. The kinetic parameters of αRβG towards rutin and hesperidin were determined in 50 mM phosphate buffer (pH 6.0) at 60 °C for 1 h, all assays were done in triplicate. The Vmax, K_M_, K_cat_ values were calculated using the Graphpad Prism software, version 8.02 (GraphPad Software, Inc). The transglycosylation reactions were performed at 30 °C. Aliphatic acceptors reactions (5% v v^−1^) were incubated 3 h. For aromatic-acceptors, 1.8 mM of the acceptor was added and incubated for 24 h. Reaction products were detected by thin-layer chromatography silica gel 60 F254 (Macherey Nagel, Sigma) using ethyl acetate/2-propanol/water (3:2:2) as mobile phase. Quantification of products were performed by hydrophilic interaction chromatography (HILIC) using photodiode array and mass spectrometry described by Fries et al. [34].

### Solubility of rutinosides

A saturated solution of resorcinol, phloroglucinol, and 4-methylumbelliferone and its rutinosides counterparts, were prepared in water and incubated at 25 °C, at 500 rpm, during 2 h. Then, the solution was centrifuged 5 min at 18,000×*g*, filtered, and analyzed by HPLC using a KONIK-500-A series HPLC system attached to a KONIK Uvis 200 detector. Samples were prepared with 30% v v^−1^ of methanol HPLC grade in miliQ water. The column was a reversed-phase LiChroCART^®^ 125–4 MERCK (12.5 cm length, 4 mm internal diameter) LiChrospher^®^ 5 µm, RP 18 (pore size 100 Å). 

Isocratic elution was performed with trifluoroacetic acid 0.1% v v^−1^ at a flow rate of 1.0 mL min^−1^ at 25 °C. The assays were performed in triplicate.

### Scale-up and purification of rutinosides

Rutinosides were enzymatically synthesized in a reaction (200 mL) containing 1.8 mM rutin, 1.8 mM Phloroglucinol or resorcinol or 4-methylumbelliferone, 0.03 U mL^−1^ of αRβG, and 2% v v^−1^ of DMSO at 30 °C incubated for 24 h. The reaction mixture was filtered with double filter paper N^o^. 103. Solvent extraction was performed twice by adding 0.5 volume of ethyl acetate. The aqueous phase was freeze dried and suspended in water. The reaction products were purified using a Biogel P2 (column 1.5 × 150 cm; flow rate of 0.1 mL min^−1^) and milliQ water as mobile phase. All fractions containing the transglycosylation products were pooled and dried by evaporation and subsequent lyophilization. 

### Cell culture and chemograms

Human Hepatocellular Carcinoma (HCC) Huh7, pancreatic cancer PANC1 and MiaPaCa-2 and pancreatic epithelial immortalized hTERT-HPNE cell lines were obtained from American Type Culture Collection (ATCC) (Manassas, VA, USA). Cell lines were grown in DMEM medium high glucose (11965092, Gibco) supplemented with 10% (v v^−1^) fetal bovine serum (FBS, 16000044, Gibco), and 1% (v v^−1^) antibiotics (5000 U mL^−1^ penicillin—5000 µg mL^−1^ streptomycin; Gibco). The hTERT-HPNE cell line was grown in DMEM medium without glucose (11966-025, Gibco), supplemented with 25% (v v^−1^) M3 Base medium (M300F-500, Incell), 5% (v v^−1^) FBS, 10 ng mL^−1^ human recombinant EGF (R&D System 236-EG) and 5.5 mM d-glucose (A24940-01, Gibco). The cultures were maintained at 37 °C in a humidified 5% CO_2_ incubator and when they reached 80–90% confluence they were routinely expanded by 0.05% trypsin detachment. For the chemograms, cells were plated in 96-wells plates with an initial concentration of 50,000 cells mL^−1^ with complete medium. After 24 h the cultures were washed and incubated in medium containing drugs alone at various concentrations or in combination with gemcitabine (100 nM) for an additional 24 h or 48 h. Cell survival was assessed by directly adding 0.5 mg mL^−1^ MTT to medium. The absorbance of the solution in each well was measured at 540 nm using an Epoch Microplate Spectrophotometer (Biotek). 

### Statistical analysis

Experiments were performed in triplicate and data were expressed as the mean ± SD of three independent experimental repetitions. Statistical analysis was performed by one- way analysis of variance (ANOVA) regarded as statistically significant and *P < 0.05. Prism Software (San Diego, CA, USA) was used to calculate IC_50_ values and their 95% confidence intervals (CI) through a nonlinear fit- curve (log of compound concentration vs normalized response–variable slope). The interaction between gemcitabine and glycoconjugates was studied using the Loewe additivity model to calculate combination index (CI) [39]. The CI values lower 1.0, equals 1 or higher 1.0 indicates synergistic, additive, or antagonistic interaction, respectively. 

## Results

### Screening for diglycosidase activity in *Acremonium* and *Sarocladium* strains

**Fig. 1:**
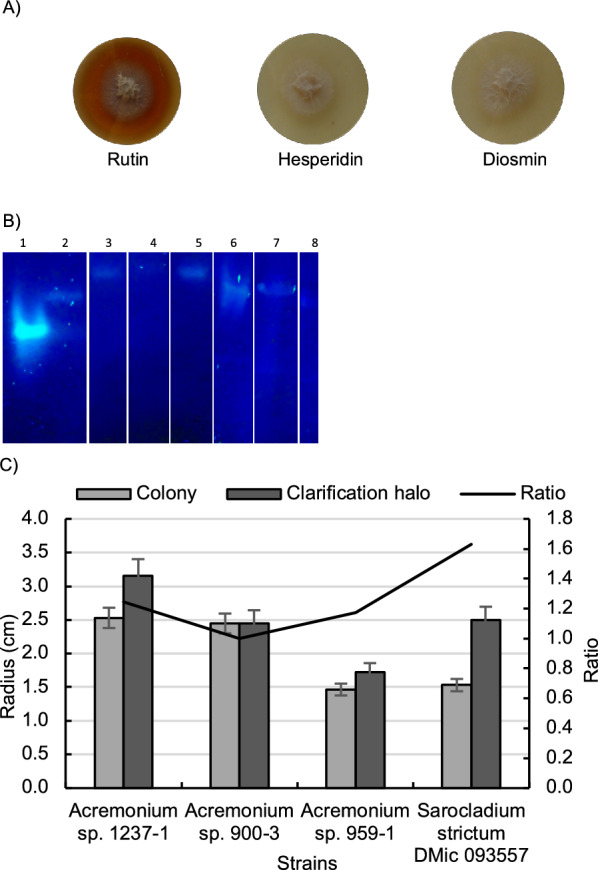
Growth and flavonoid degradation of *Acremonium* and *Sarocladium* strains. **A** *Acremonium* sp. 1237-1 grown on agar using rutin, hesperidin or diosmin as carbon sources for 10 days and 25 °C. Rutin plate showing zone of flavonoid hydrolysis. **B** Zymographic analysis of extracellular extracts using 4-methylumbelliferyl-rutinoside as substrate. Lane 1: 6-*O*-α-l-rhamnosyl-β-d-glucosidase (EC 3.2.1.168) from *Acremonium* sp. DSM24697; Lane 2: *S. strictum* DMic 093557; Lane 3: *S. strictum* DMic 993190; Lane 4: *S. strictum* DMic 114098; Lane 5: *S. kiliense* DMic 00226; Lane 6: *Acremonium* sp. 959-1; Lane 7: *Acremonium* sp. 1237-1; Lane 8: *Acremonium* sp. 900-3. **C** Strains selection for flavonoid degradation considering ratio between clarification halo and colony diameter

Initially, we evaluated diglycosidase activity in twenty-eight fungal strains of the genera *Acremonium* and *Sarocladium* using the flavonoids rutin, diosmin and hesperidin as carbon source. These genera were selected based on their recognition as producers of structurally diverse and pharmacologically active compounds [40]. Flavonoids inhibited the growth of *Acremonium* sp. 147-1 and *S. kiliense* DMic 062925, however the rest of tested strains grew in the presence of these molecules (Additional file 1: Table S1). The clarification halo in the solid culture, indicating flavonoid degradation, was detected in 21 strains growing on rutin, while it was undetected on the media containing hesperidin and diosmin as carbon sources (Fig. 1A; Additional file 1: Table S1). Diglycosidases, contrary to exoglycosidases, do not hydrolyze diglycosides by the stepwise release of monosaccharides in two sequential reactions; instead, they specifically hydrolyze the glycosidic bond between the disaccharide and the aglycone [41]. Since diglycosidases compete for their substrates with exoglycosidases, it is important to distinguish these two types of activities. To this end, a zymographic assay with 4-methylumbelliferyl-rutinoside as a substrate was used to confirmed diglycosidase activity. The assay revealed a single positive band in seven of the strains (*Acremonium* sp. 959-1, 1237-1, 900-3, *S. strictum* DMic 093557, DMic 993190, DMic 114098 and *S. kiliense* DMic 00226) (Fig. 1B). Growth parameters such as radial growth (*K*r) and clarification rates (*K*c) were calculated with rutin throughout twenty days for *Acremonium* sp. 1237-1, 959-1, 900-3 and *S. strictum* DMic 093557 (Table 1).

**Table 1:** Growth and clarification rates of *Acremonium* sp. 1237-1, *Acremonium* sp. 959-1, *Acremonium* sp. 900-3 and *S. strictum DMic 093557*

Strain	Kr (mm day^−1^)	Kc (mm day^−1^)
*Acremonium* sp. 1237-1	1.32	1.04
*Acremonium* sp. 900-3	1.31	0.71
*Acremonium* sp. 959-1	0.72	0.67
*Sarocladium* DMic 093557	0.85	2.01

Considering ratio between the colony and clarification halo diameter, *S. strictum* DMic 093557 was the selected strain for further characterization (Fig. 1C).

### Characterization of the rutin deglycosylation and transglycosylation activity of *S. strictum*

**Fig. 2:**
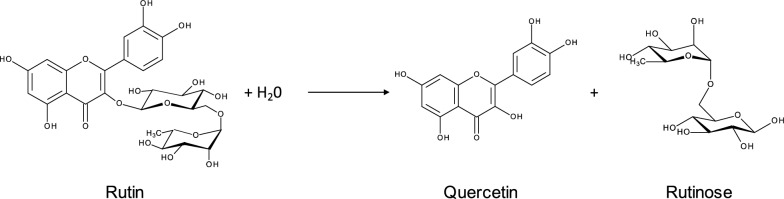
Reaction scheme of the rutin hydrolysis catalyzed by 6-*O*-α-l-rhamnosyl-β-d-glucosidase from *S. strictum*

Submerged cultures of *S. strictum* with rutin as carbon source produced the maximum deglycosylation activity (22.4 ± 4.1 U L^−1^) after 8 days at 28 °C. Rutinose and quercetin were the main reaction products of rutin hydrolysis, confirming that deglycosylation is through diglycosidase activity (Fig. 2).

**Fig. 3:**
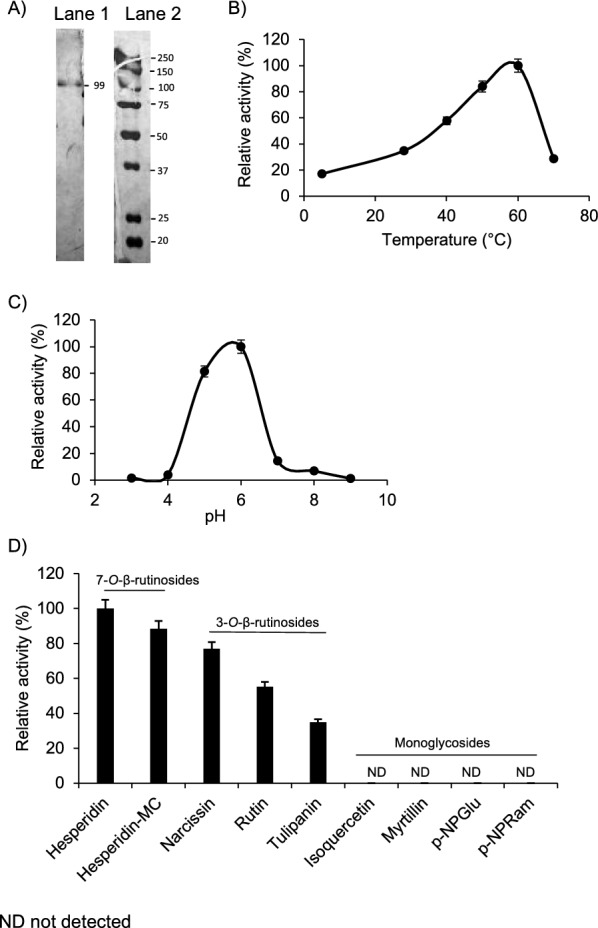
Biochemical characterization of αRβG from *S. strictum* DMic 093557. **A** Enzyme purification to homogeneity: Lane 1. αRβG, Lane 2. Molecular mass marker. **B** Temperature profile of αRβG, (50 mM sodium phosphate buffer pH 6.0, 100% activity corresponded to 0.7 U mL^−1^). **C** pH profile of αRβG at 60 °C (100% activity corresponded to 0.3 U mL^−1^) **D** Substrate specificities of αRβG (100% activity corresponded to 0.1 U mL^−1^)

The enzyme 6-*O*-α-rhamnosyl-β-glucosidase (αRβG) was purified 3.88 folds using biomass filtration, ammonium sulfate precipitation and size-exclusion chromatography with 4.08% yield (Additional file 1: Table S2). The molecular mass of 99 kDa was estimated by SDS-PAGE and tryptic peptide sequences were obtained by MALDI-TOF mass spectrometry (Fig. 3A). The peptide TIHETYLAPWYDGVK was related to the putative glycoside hydrolase gene (2275 bp) of 755 amino acids, including a secretory signal peptide of 21 residues at the N-terminus of the strain *A. strictum* DS1bioAY4a (ID, 1029425) (https://genome.jgi.doe.gov) (Additional file 1: Fig. S1). The enzyme exhibits activity at a range of temperatures between 30 and 60 °C with a maximum at 60 °C and markedly declines at 70 °C (Fig. 3B). The optimal pH was 6.0, exhibiting more than 60% relative activity in the range of pH 4.5–pH 6.5 (Fig. 3C). Substrate specificity of αRβG was evaluated using artificial substrates and glycosylated flavonoids. αRβG exhibited higher hydrolytic activity toward the 7-*O*-β-rutinosylated flavonoids, hesperidin and hesperidin-methylchalcone, followed by 3-*O-*β-rutinosylated flavonoids narcissin, rutin (quercetin 3-*O*-rutinoside) and tulipanin (delphinidin 3-*O*-rutinoside) (Fig. 3D). Interestingly, αRβG did not show hydrolytic activity with the monoglycosylated flavonoids isoquercetin (quercetin 3-*O*-β-glucoside) and myrtillin (delphinidin 3-*O*-glucoside). Furthermore, the catalyst was not able to release the glycosidic residue from the artificial substrates, *p*-Nitrophenyl-β-d-glucopyranoside and *p*-Nitrophenyl-α-l-rhamnopyranoside (Fig. 3D). These results demonstrate a high promiscuity of the biocatalyst for rutinosides compounds and the undetectable activity onto monoglycosylated substrates (Fig. 3D).

The *K*_M_ and *k*_cat_ values of αRβG were determined for the flavonoids rutin and hesperidin (Table 2) and the enzyme exhibited typical Michaelis–Menten kinetics. The *K*_M_ values were 0.473 ± 0.004 and 0.70 ± 0.12 mM for rutin and hesperidin, respectively (Table 2). While, the *k*_cat_ values for rutin was 0.53 ± 0.06 and for hesperidin was 0.94 ± 0.11 s^−1^, indicating a higher efficiency for hesperidin hydrolysis than rutin hydrolysis (hesperidin *k*_cat_ > 1.77-fold) (Table 2).

**Table 2:** Kinetic constants of αRβG from *S. strictum DMic 093557*

Substrate	K_M_ [mM]	k_cat_ [s^−1^]	k_cat_/K_M_ [s^−1^ mM^−1^]
Hesperidin	0.70	0.94	1.34
Rutin	0.47	0.53	1.12

**Fig. 4:**
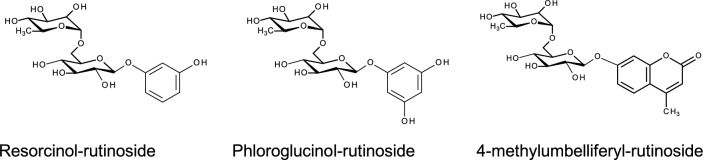
Chemical structures of rutinose- derivatives

Next, to determine the synthesis capability of αRβG, rutin was used as rutinose donor and several compounds as acceptors. Transglycosylation activity was not detected in primary alcohols with short- chain while those that contain 4 or more carbons (butanol, isoamyl alcohol and 2-phenylethanol) exhibited the highest yields (Table 3). The lowest transglycosylation yield for αRβG was determined with the secondary alcohol isopropanol. Regarding phenolic acceptors, αRβG was able to transfer the rutinosyl moiety to phloroglucinol, resorcinol and 4-methylumbelliferone with considerable yields to obtain, respectively rutinosides (Table 3; Fig. 4). The fact that αRβG could transfer the disaccharide unit to aliphatic and phenolic alcohols with acceptable yields, indicate the interesting biosynthetic potential of this biocatalysts.

**Table 3:** Tranglycosylation yields of αRβG from *S. strictum* DMic 093557 with different OH-acceptors

Acceptor	Yield (mM)	Acceptor	Yield (mM)
Aliphatic		Aromatic	
Isoamyl alcohol	0.62	Phloroglucinol	0.38
Butanol	0.47	4-methylumbelliferone (4MU)	0.37
2-phenylethanol	0.39	Resorcinol	0.36
*n*-propanol	0.26		
Isopropanol	0.20		
Ethanol	ND		
Methanol	ND		

*ND* not detected

To confirm the improvement of solubility of novel compounds, the aqueous solubility of each glycoside at room temperature was compared with that of the respective aglycon. Solubility detected for resorcinol was of 0.94 g mL^−1^, and the glycoside had its solubility increased up to 1.93 g mL^−1^. 4-methylumbelliferone was insoluble in aqueous solution and 4MUR solubility was 0.13 g mL^−1^. Phloroglucinol showed a solubility of 0.01 g mL^−1^, which was increased by transglycosylation to a value of 0.097 g mL^−1^. This data confirmed the augmented of the solubility of the novel glycodrugs, making them more bio-available.

### Optimization of rutinosides synthesis

**Fig. 5:**
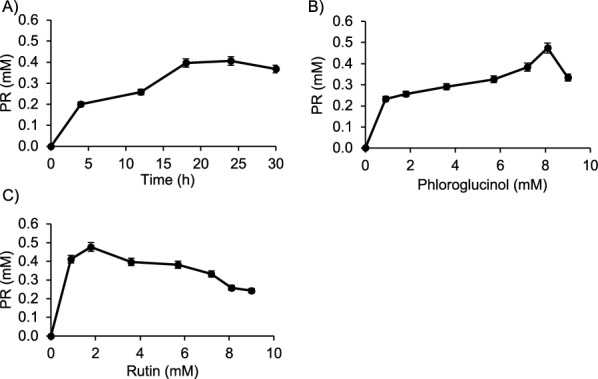
Optimization of phloroglucinol-rutinoside production using αRβG from *S. strictum* DMic 093557. **A** Time course using 0.05 U mL^−1^. **B** Effect of phloroglucinol concentration on the enzymatic synthesis of phloroglucinol-rutinoside at 24 h. **C** Effect of rutin concentration on the enzymatic synthesis of pholroglucinol-rutinoside using 8.1 mM phloroglucinol at 24 h

Since phloroglucinol exhibited cell growth inhibition against cancer cells [42–44] and displayed the highest transglycosylation yield within the OH-phenolic substrates assayed, one-pot synthesis and scale up of PR was carried out using rutin as sugar donor. Time course showed the highest production of the rutinoside compound (0.40 mM) after 24 h reaction, achieving a yield of 21.1% w w^−1^ regarding the substrate rutin (Fig. 5A). Afterward, the yield decreased indicating that the formed PR was hydrolyzed by the enzyme. The optimal concentration of the OH-acceptor (phloroglucinol) was 8.1 mM (Fig. 5B). Regarding sugar donor concentration, PR yield increased with rutin concentration up to 1.8 mM (Fig. 5C). Over this concentration, the reaction efficiency was reduced possibly because of low solubility of rutin in the aqueous medium. Optimization of the reaction conditions resulted in an improved yield of 23%. The established conditions were used for the glycosylation of resorcinol and 4-methylumbelliferone considering these bioactive compounds were also reported to have anti-proliferative activity [45–48]. The same conditions were applied for production up to milligrams scale.

### Evaluation of the cytotoxicity activities of rutinosides derivatives

**Fig. 6:**
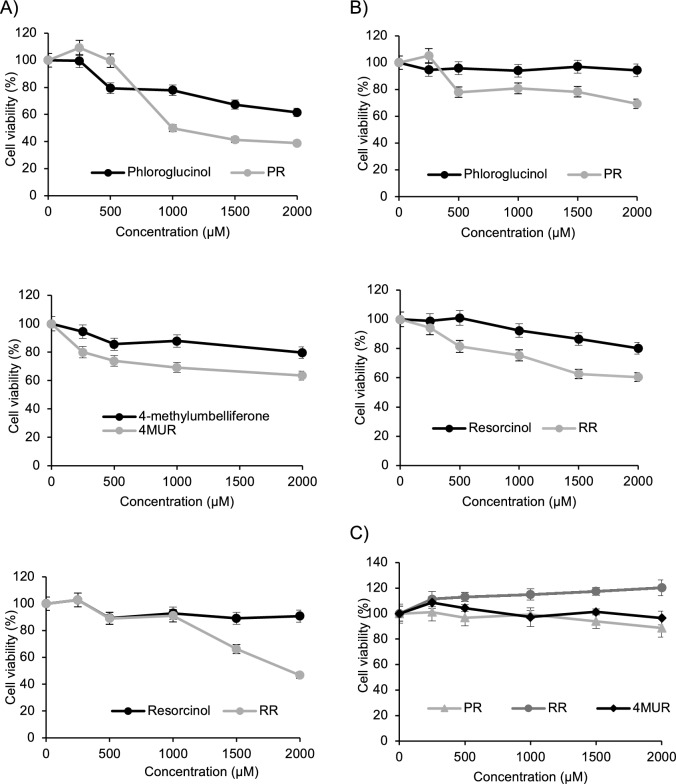
Chemograms assays showing the sensitivity on liver and pancreatic cells to resorcinol, phloroglucinol and 4-methylumbelliferone and their respective rutinosides. Dose–response curves for cell lines (**A**) PANC1 (**B**) Huh7 (**C**) Effect of RR, PR and 4MUR on hTERT-HPNE cell line. The concentrations of each drug vary from 0.25 mM to 2.0 mM. The results correspond to three independent experiments expressed as the mean ± SD proliferation average (*P < 0.05)

Next, we sought to investigate the potential therapeutic activity of the synthetized PR, RR and 4MUR and their respective aglycones used human liver and pancreas tumor cell lines, Huh7 and PANC1, respectively. Chemograms were performed using increasing concentrations (0–2.0 mM) of the compounds during 48 h. In PANC1, PR showed the highest effectiveness with an IC_50_ of 0.89 mM, RR exhibited an IC_50_ of 1.67 mM and 4MUR revealed an IC_50_ of 2.4 mM (Fig. 6A). In Huh7 cells the glycosylated compounds produced lower cytotoxic effect, PR (3.8 mM) and RR (4.5 mM) were effective in reducing growth by > 50% (Fig. 6B). While the effect 4MUR on Huh7 cells was previously reported by our group [22]. It is worth mentioning that in both cell lines glycosylated compounds exhibited stronger anti-proliferative effects when compared to their aglycone counterparts (Fig. 6). Based on these results, we evaluate the specificity of the effect of RR, PR and 4MUR on hTERT-HPNE epithelial cells of pancreatic origin. Notably, none compounds affected cell viability (IC_50_ > 8 mM) (Fig. 6C). These finding provide initial evidence for the improves anti-tumoral effect of glycoconjugates per se using a dose that had no effect on normal pancreatic epithelial cells.

**Fig. 7:**
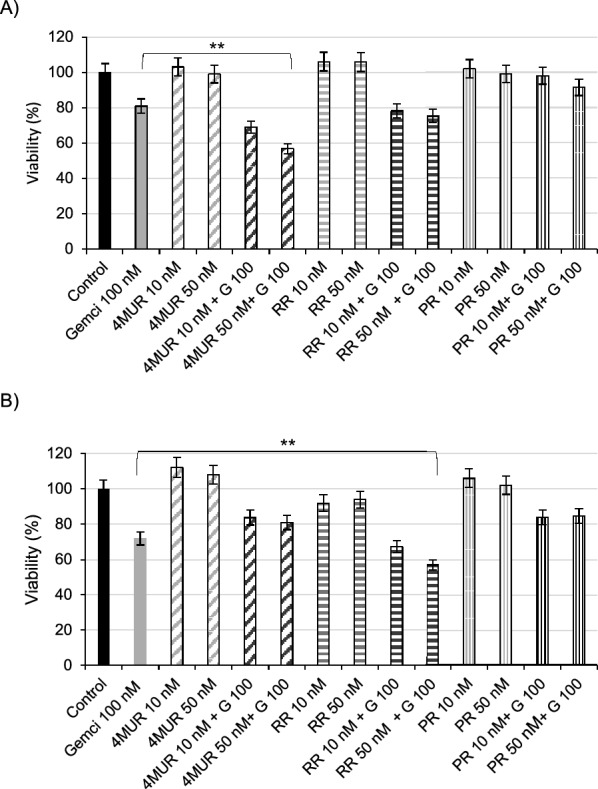
Sensitizing effect of rutinosides to gemcitabine in pancreatic cancer cells after 24 h treatment. **A** PANC1 and (**B**) MiaPaCa-2 cells treated with 100 nM gemcitabine (G 100) and 4-methylumbelliferyl-rutinoside (4MUR), resorcinol-rutinoside (RR) and phloroglucinol-rutinoside (PR) at 10 and 50 nM. The results correspond to three independent experiments expressed as the mean ± SD proliferation average (*P < 0.05)

Subsequent studies were undertaken to examine whether pancreatic cancer cells co-treated with rutinosides could be more sensitive to gemcitabine. Notable, in gemcitabine-resistant PANC1 cells, 4MUR showed an anti-neoplastic effect decreasing the cell viability 44% (100 nM Gemcitabine, 50 nM 4MUR, 48 h, p < 0.05) (Fig. 7A). While in gemcitabine-sensitive MiaPaCa-2 cells, RR caused 42% loss of viable cells (100 nM Gemcitabine with100 nM RR, 48 h, p < 0.05) (Fig. 7B).

To evaluate the nature of drug interactions, CI was calculated for each combination of gemcitabine 100 nM and glycoconjugates at 10 and 50 nM. In gemcitabine- sensitive MiaPaCa-2 cells, our findings reveal that 4MUR exhibit an additive interaction at both 10 and 50 nM concentrations. Additionally, RR at 10 nM displays an additive interaction, while at 50 nM, exhibits a synergistic interaction. In the gemcitabine-resistant cell line PANC1, both RR and 4MUR, across the studied concentrations, display synergistic interactions with gemcitabine at 100 nM (Table 4).

**Table 4:** Combination index (CI) for gemcitabine with 4MUR, RR or PR in PANC1 and MiaPaCa-2 cells

Combination	CI	
	PANC1	MiaPaCa-2
4MUR 10 nM + G 100 nM	0.82	1.03
4MUR 50 nM + G 100 nM	0.71	1.04
RR 10 nM + G 100 nM	0.91	1.01
RR 50 nM + G 100 nM	0.87	0.84
PR 10 nM + G 100 nM	1.18	1.10
PR 50 nM + G 100 nM	1.14	1.15

## Discussion

In this work, using a panel of *Acremonium* and *Sarocladium* fungi we found a subset of strains capable of growing in rutin as carbon source and exhibiting diglycosidase activity. The enzyme produced by *S. strictum* hydrolyses 7-*O*-rutinosides (hesperidin, heperidin-methylchalcone) and 3-*O*-rutinosides (rutin, narcisin, tulipanin) as most of fungal diglycosidases reported [25, 27, 49, 50]. It is interesting to point out, that the majority of diglycosidases reported displayed activities with both mono- and di-glycosylated substrates [25, 27, 49, 50]. These broad substrate specificities make difficult to classify them as β-glucosidases or β-rutinosidases. Kotik et al., reported the relevance of conserved aromatic aminoacids in the site + 1 of the biocatalysts active site that might explain this enzymatic feature [51]. In contrast, *S. strictum* αRβG presented null hydrolytic activity with any of mono-glycosylated compounds tested. This particular behaviour differentiated αRβG from others enzymes reported and can clearly be defined as a diglycosidase.

Synthesis of a series of glycosides has been reported using diglycosidases [23, 26, 29]. However, the addition of rutinose moiety to phenolic acceptors is a rare event; for a long time, it had been believed that glycosidases employing a retaining mechanism were unable to glycosylate phenolic phenolic OH. Up to now, few fungal diglycosidases were able to transglycosylate aromatic alcohols and usually, yields of transglycosylation reaction were reduced by the increment of the bulkiness of the acceptor [23, 26, 29]. The transglycosylation products of *S. strictum* DMic 093557 αRβG with methanol and ethanol were not detected, while the highest yield of transglycosylation was reached with longer carbon chain alcohols such as isoamylalcohol. Furthermore, αRβG demonstrates transglycosylation activity with phenolic acceptors such as phloroglucinol, resorcinol, and 4-methylumbeliferone. This underscores the significant potential of αRβG as a biocatalyst for producing rutinosides compounds with pharmacological applications. 

The biomedical importance of these glycosylated molecules stems primarily from the presence of the rhamnose moiety, one of the monosaccharide units that form rutinose. Notably, the absence of genes for rhamnose synthesis and rhamnosidases in mammals ensures the robust stability of rhamnosylated glycoconjugates in vivo [52]. Lou’s group demonstrate that rhamnose moiety plays a critical role in the anti-tumor effect of solasodine-derived rhamnosides [53]. This effect is in part due to the interaction between rhamnose and rhamnose binding lectin receptors (RBL) located on the surface of tumor cells facilitating rhamnosides derivatives cellular uptake. The specific targeting of rhamnose-capped drugs to the RBL over-expressed in breast cancer cells was also confirmed by Xu et al. [54]. However, rhamnoside derivatives drugs did not exhibit significant differences in comparison with the cytotoxic effects of the parental drug. Furthermore, the Liu’s group reported that while conjugating one rhamnose residue to emodin increased the water solubility of the derivative, it did not alter its anti-cancer activity [55]. However, emodin rhamnosylated derivatives with more complex sugar chains in their structure exhibited a significant enhancement in anti-proliferative activity. Recently, our group reported that 4MUR, which carries a disaccharidic unit and could be considered a complex-sugar drug was more effective than the aglycone 4-methylumbelliferone in HCC models [22]. The enhanced complexity in the sugar chains attached to bioactive drugs seems to contribute to the overall efficacy of these compounds against cancer. Previous results in hepatocellular carcinoma suggest that glycosylated compound have increased cellular uptake. This can be attributed to the involvement of asialoglycoprotein receptor (ASGPRs) in the internalization of glycoconjugates bearing exposed galactose, rhamnose, or *N*-acetylgalactosamine residues. This was further supported by results identifying free rutinose as a competitive ligand capable of impeding the binding of 4MUR to ASGPRs, and consequently diminishing its cellular uptake [22].

Current therapies for the treatment of pancreatic cancer have shown disappointing efficacy. Thus, there is an imperative need to develop novel strategies to improve the patient’s outcome. Bioactive compounds that selectively inhibit molecular pathways involved in the growth and survival of tumor cells may represent an effective or complementary alternative to standard cancer chemotherapeutic drugs contributing to increasing the efficacy of the treatment [56–59]. Previous studies have reported the anti-proliferative properties of certain bioactive compounds, including phloroglucinol, resorcinol, and 4-methylumbelliferone, across various cancer types (42–48). Our results demonstrate that sugar-modified compounds RR, PR y 4MUR have a higher growth inhibitory potency in pancreatic cancer cells compared to aglycone counterpart. It is worth noting that potent anti-cancer drugs typically exhibit IC_50_ values in the low micromolar (µM) to nanomolar (nM) range. However, the cytotoxicity activities of rutinoside derivatives are positioned within the range expected for early phases of drug development. Furthermore, while it is true that IC_50_ values in mM levels may initially appear to suggest lower potency, they also hint at a wider therapeutic window. This implies a potentially lower risk of adverse effects or toxicity, enhancing the safety profile of these compounds for therapeutic applications. Interestingly, rutinose-conjugates did not exhibit significant effect on the growth of pancreatic epithelial cells, underlining their selective cytotoxicity against pancreatic cancer cells while not affecting normal pancreatic cells growth. Furthermore, enhanced the sensitivity in pancreatic tumour cells to the standard treatment gemcitabine across both, gemcitabine-resistant and gemcitabine-sensitive cell lines. These findings suggest rutinosides as a promising therapeutic approach for pancreatic cancer.

## Conclusions

A disaccharide unit was transferred throughout an enzymatic glycosylation using the diglycosidase αRβG from *S. strictum* DMic 093557. The synthetized rutinosides exhibited higher water solubility and enhanced anti-proliferative activities compared to the aglycone counterpart, offering new avenues for the development of new treatment approaches for cancer patients. The αRβG transglycosylate-based system also provides a platform for optimizing the pharmacological properties of bioactive molecules, thereby paving the way for the design and implementation of more effective therapeutic strategies for pancreatic cancer and other neoplasms.

### Supplementary Information

**Additional file 1: Table S1. **a) Growth of *Acremonium *and *Sarocladium *strains with the flavonoids diosmin, herperidin and rutin as carbon source and b) clarification halo of rutin detection. **Table S2.** Purification of α-rhamnosyl-β-glucosidase from *Sarocladium strictum *DMic 093557. **Fig. S1.** Protein sequence of the glycoside hydrolase from *Acremonium strictum* DS1bioAY4a including predicted signal peptide. The determined tryptic peptide sequence is shown after alignment.

## Data Availability

Not applicable.

## Abbreviations

4MUR: 4-Methylumbelliferyl-rutinoside
ANOVA: Analysis of variance
ATCC: American Type Culture Collection
BSA: Bovine serum albumin
EGF: Epidermal Growth Factor
FBS: Fetal bovine serum
FDA: U.S Food and Drug Administration
GHs: Glycoside hydrolases
GTs: Glycosyltransferases
HCC: Hepatocellular carcinoma
HILIC: Hydrophilic interaction chromatography
HPLC: High performance liquid chromatography
LB: Luria–Bertani medium
MTT: 3-(4,5-Dimethylthiazol-2-yl)-2,5-Diphenyltetrazolium Bromide assay
PR: Phloroglucinol-rutinoside
RBL: Receptor rhamnose-binding lectin
RR: Resorcinol-rutinoside
αRβG: 6-*O*-α-Rhamnosyl-β-glucosidase

## References

[1] Stuurman FE, Nuijen B, Beijnen JH, Schellens JHM (2013). Oral anticancer drugs: mechanisms of low bioavailability and strategies for improvement. Clin Pharmacokinet.

[2] Ioele G, Chieffallo M, Occhiuzzi MA, Ragno G, Grande F, De LM (2022). Pharmacokinetic and pharmacodynamic properties. Molecules.

[3] Iglesias LE, Lewkowicz ES, Medici R, Bianchi P, Iribarren AM (2015). Biocatalytic approaches applied to the synthesis of nucleoside prodrugs. Biotechnol Adv.

[4] Agarwal R, Agarwal C, Ichikawa H, Singh RP, Aggarwal BB (2006). Anticancer potential of silymarin: from bench to bed side. Anticancer Res.

[5] Martin H, Lázaro LR, Gunnlaugsson T, Scanlan EM (2022). Glycosidase activated prodrugs for targeted cancer therapy. Chem Soc Rev.

[6] Molejon MI, Weiz G, Breccia JD, Vaccaro MI (2020). Glycoconjugation: an approach to cancer therapeutics. World J Clin Oncol.

[7] Chen F, Huang G (2019). Application of glycosylation in targeted drug delivery. Eur J Med Chem.

[8] Cai L, Gu Z, Zhong J, Wen D, Chen G, He L (2018). Advances in glycosylation-mediated cancer-targeted drug delivery. Drug Discov Today.

[9] Shaul P, Steinbuch KB, Blacher E, Stein R, Fridman M (2015). Exploring the effects of glycosylation and etherification of the side chains of the anticancer drug mitoxantrone. ChemMedChem.

[10] Biocatalysis S, Pyser JB, Chakrabarty S, Romero EO, Narayan ARH (2021). State-of-the-art biocatalysis. ACS cent Sci.

[11] Wu S, Snajdrova R, Moore JC, Baldenius K, Bornscheuer UT (2021). Biocatalysis: enzymatic synthesis for industrial applications. Angew Chem Int Edition.

[12] Danby PM, Withers SG (2016). Advances in enzymatic glycoside synthesis. ACS Chem Biol.

[13] Malik A, Seeberger PH, Varón SD (2021). Advances in the chemical synthesis of carbohydrates and glycoconjugates. Adv Biochem Eng Biotechnol.

[14] Andreotti G, Trincone A, Giordano A (2007). Convenient synthesis of β-galactosyl nucleosides using the marine β-galactosidase from Aplysia fasciata. J Mol Catal B Enzymatic..

[15] Singh S, Aggarwal A, Dinesh NVS, Bhupathiraju K, Arianna G, Tiwari K, Drain CM (2015). Glycosylated porphyrins, phthalocyanines, and other porphyrinoids for diagnostics and therapeutics. Chem Rev.

[16] Yan LQ, Li N, Zong MH (2012). First and facile enzymatic synthesis of β-fucosyl-containing disaccharide nucleosides through β-galactosidase-catalyzed regioselective glycosylation. J Biotechnol.

[17] Desmet T, Soetaert W, Bojarová P, Křen V, Dijkhuizen L, Eastwick-Field V, Schiller A (2012). Enzymatic glycosylation of small molecules: challenging substrates require tailored catalysts. Chem Eur J.

[18] Ati J, Lafite P, Daniellou R (2017). Enzymatic synthesis of glycosides: from natural *O*- and *N*-glycosides to rare *C*- and *S*-glycosides. Beilstein J Org Chem.

[19] Zuzana M, Nekvasilov P, Bojarov P, Vladimír K (2021). Advanced glycosidases as ingenious biosynthetic instruments. Biotechnol Adv.

[20] Withers SG (2001). Mechanisms of glycosyl transferases and hydrolases. Carbohydr Polym.

[21] Křen V, Bojarová P. Rutinosidase and other diglycosidases: rising stars in biotechnology. Biotechnol Adv. 2023; 108217. 10.1016/j.biotechadv.2023.108217 37481095

[22] Weiz G, Molejon MI, Malvicini M, Sukowati CHC, Mazzolini G, Breccia JD (2022). Glycosylated 4-methylumbelliferone as a targeted therapy for hepatocellular carcinoma. Liv Int.

[23] Haluz P, Kis P, Cvečko M, Mastihubová M, Mastihuba V (2023). Acuminosylation of tyrosol by a commercial diglycosidase. Int J Mol Sci.

[24] Bassanini I, Kapešová J, Petrásková L, Pelantová H, Markošová K, Rebroš M (2019). Glycosidase-catalyzed synthesis of glycosyl esters and phenolic glycosides of aromatic acids. Adv Synth Catal.

[25] Šimčíková D, Kotik M, Weignerová L, Halada P, Pelantová H, Adamcová K (2014). α-l-Rhamnosyl-β-d-glucosidase (rutinosidase) from *Aspergillus niger*: characterization and synthetic potential of a novel diglycosidase. Adv Synth Catal.

[26] Mazzaferro LS, Weiz G, Braun L, Kotik M, Pelantová H, Křen V (2019). Enzyme-mediated transglycosylation of rutinose (6-*O*-α-l-rhamnosyl-d-glucose) to phenolic compounds by a diglycosidase from *Acremonium* sp. DSM 24697. Biotechnol Appl Biochem.

[27] Weiz G, Mazzaferro LS, Kotik M, Neher BD, Halada P, Křen V (2019). The flavonoid degrading fungus *Acremonium* sp. DSM 24697 produces two diglycosidases with different specificities. Appl Microbiol Biotechnol.

[28] Giraldo A, Gené J, Sutton DA, Madrid H, de Hoog GS, Cano J (2015). Phylogeny of *Sarocladium* (Hypocreales). Pers Mol Phylogeny Evol Fungi.

[29] Mazzaferro LS, Piñuel L, Erra-Balsells R, Giudicessi SL, Breccia JD (2012). Transglycosylation specificity of *Acremonium* sp. α-rhamnosyl-β-glucosidase and its application to the synthesis of the new fluorogenic substrate 4-methylumbelliferyl-rutinoside. Carbohydr Res.

[30] González C, Martinez A, Vázquez F, Baigorí M, de Figueroa LIC (1996). New method of screening and differentiation of exoenzymes from industrial strains. Biotechnol Tech.

[31] Laemmli U (1970). Cleavage of structural proteins during the assembly of the head of bacteriophage T4. Nature.

[32] Miller GL (1959). Use of dinitrosaiicyiic acid reagent for determination of reducing sugar. Anal Chem.

[33] Weiz G, Breccia JD, Mazzaferro LS (2017). Screening and quantification of the enzymatic deglycosylation of the plant flavonoid rutin by UV–visible spectrometry. Food Chem.

[34] Fries AA, Mazzaferro L, Grüning B, Stibal K, Buchholz P, Pleiss J (2019). Alteration of the route to menaquinone towards isochorismate-derived metabolites. ChemBioChem.

[35] Candiano G, Bruschi M, Musante L, Santucci L, Ghiggeri GM, Carnemolla B (2004). Blue silver: a very sensitive colloidal Coomassie G-250 staining for proteome analysis. Electrophoresis.

[36] Artimo P, Jonnalagedda M, Arnold K, Baratin D, Csardi G, De Castro E (2012). ExPASy: SIB bioinformatics resource portal. Nucleic Acids Res.

[37] Petersen TN, Brunak S, Von Heijne G, Nielsen H (2011). SignalP 4.0: discriminating signal peptides from transmembrane regions. Nat Methods.

[38] Smith PK, Krohn RI, Hermanson GT, Mallia AK, Gartner FH, Provenzano MD (1985). Measurement of protein using bicinchoninic acid. Anal Biochem.

[39] Loewe S, Muchnik H (1926). Effect of combinations: mathematical basis of problem. Arch Exp Pathol Pharmacol.

[40] Qin Y, Lu H, Qi X, Lin M, Gao C (2024). Recent advances in chemistry and bioactivities of secondary metabolites from the genus *Acremonium*. J Fungi.

[41] Mazzaferro LS, Breccia JD (2011). Functional and biotechnological insights into diglycosidases*. Biocatal Biotransform.

[42] Pádua D, Rocha E, Gargiulo D, Ramos AA (2015). Bioactive compounds from brown seaweeds: phloroglucinol, fucoxanthin and fucoidan as promising therapeutic agents against breast cancer. Phytochem Lett.

[43] Kim RK, Uddin N, Hyun JW, Kim C, Suh Y, Lee SJ (2015). Novel anticancer activity of phloroglucinol against breast cancer stem-like cells. Toxicol Appl Pharmacol.

[44] Kim RK, Suh Y, Yoo KC, Cui YH, Hwang E, Kim HJ (2015). Phloroglucinol suppresses metastatic ability of breast cancer cells by inhibition of epithelial–mesenchymal cell transition. Cancer Sci.

[45] Shahinozzaman M, Ishii T, Halim MA, Hossain MA, Islam MT, Tawata S (2019). Cytotoxic and anti-inflammatory resorcinol and alkylbenzoquinone derivatives from the leaves of *Ardisia sieboldii*. Z Naturforsch C J Biosci.

[46] Pibuel MA, Poodts D, Díaz M, Molinari YA, Franco PG, Hajos SE (2021). Antitumor effect of 4MU on glioblastoma cells is mediated by senescence induction and CD44, RHAMM and p-ERK modulation. Cell Death Discov.

[47] Rodríguez MM, Fiore E, Bayo J, Atorrasagasti C, García M, Onorato A (2018). 4Mu decreases CD47 expression on hepatic cancer stem cells and primes a potent antitumor T cell response induced by interleukin-12. Mol Ther.

[48] Nagase H, Kudo D, Suto A, Yoshida E, Suto S, Negishi M (2017). 4-Methylumbelliferone suppresses hyaluronan synthesis and tumor progression in SCID mice intra-abdominally inoculated with pancreatic cancer cells. Pancreas.

[49] Ishikawa M, Kawasaki M, Shiono Y, Koseki T (2018). A novel *Aspergillus oryzae* diglycosidase that hydrolyzes 6-*O*-α-l-rhamnosyl-β-d-glucoside from flavonoids. Appl Microbiol Biotechnol.

[50] Mazzaferro L, Piñuel L, Minig M, Breccia JD (2010). Extracellular monoenzyme deglycosylation system of 7-*O*-linked flavonoid β-rutinosides and its disaccharide transglycosylation activity from *Stilbella fimetaria*. Arch Microbiol.

[51] Kotik M, Jav H, Brodsky K, Pelantová H (2021). Two fungal flavonoid-specific glucosidases/rutinosidases for rutin hydrolysis and rutinoside synthesis under homogeneous and heterogeneous reaction conditions. AMB Express.

[52] Actis- Goretta L, Dew TP, Lévèques A (2015). Gastrointestinal absorption and metabolism of hesperetin-7-*O*-rutinoside and hesperetin-7-*O*-glucoside in healthy humans. Mol Nutr Food Res.

[53] Wang Y, Gao J, Gu G, Li G, Cui C, Sun B (2011). In situ RBL receptor visualization and its mediated anticancer activity for solasodine rhamnosides. ChemBioChem.

[54] Xu L, Liu X, Li Y, Yin Z, Jin L, Lu L (2019). Enzymatic rhamnosylation of anticancer drugs by an α-l-rhamnosidase from *Alternaria* sp. L1 for cancer-targeting and enzyme-activated prodrug therapy. Appl Microbiol Biotechnol.

[55] Xing JY, Song GP, Deng JP, Jiang LZ, Xiong P, Yang BJ (2015). Antitumor effects and mechanism of novel emodin rhamnoside derivatives against human cancer cells in vitro. PLoS ONE.

[56] Pandita A, Kumar B, Manvati S, Vaishnavi S, Singh SK, Bamezai RNK (2014). Synergistic combination of gemcitabine and dietary molecule induces apoptosis in pancreatic cancer cells and down regulates PKM2 expression. PLoS ONE.

[57] Majhi S (2023). Recent developments in the synthesis and anti-cancer activity of acridine and xanthine-based molecules. Phys Sci Rev..

[58] Majhi S (2022). Discovery, development and design of anthocyanins-inspired anticancer agents: a comprehensive review. Anticancer Agents Med Chem.

[59] Majhi S, Das D (2021). Chemical derivatization of natural products: semisynthesis and pharmacological aspects—a decade update. Tetrahedron.

