# Supplementary material for: Rutinosides-derived from Sarocladium strictum 6-O-α-rhamnosyl-β-glucosidase show enhanced anti-tumoral activity in pancreatic cancer cells
Source: Microb Cell Fact. 2024 May 8;23:133. doi: 10.1186/s12934-024-02395-0 (PMC11077868; doi:10.1186/s12934-024-02395-0)
Supplement: Supplementary file 1 — Additional file 1: Table S1. a) Growth of Acremonium and Sarocladium strains with the flavonoids diosmin, herperidin and rutin as carbon source and b) clarification halo of rutin detection. Table S2. Purification of α-rhamnosyl-β-glucosidase from Sarocladium strictum DMic 093557. Fig. S1. Protein sequence of the glycoside hydrolase from Acremonium strictum DS1bioAY4a including predicted signal peptide. The determined tryptic peptide sequence is shown after alignment. [file 12934_2024_2395_MOESM1_ESM.pdf]

Additional file

Rutinosides-derived from *Sarocladium strictum* 6-*O*-α-rhamnosyl-β-glucosidase show a novel anti-tumoral activity in pancreatic cancer

Gisela Weiz*[1]; Alina L. González*[1]; Iara S. Mansilla[1];
Martín E. Fernandez-Zapico[2]; María I. Molejón[1]; Javier D. Breccia[1]

[1] Facultad de Ciencias Exactas y Naturales, Instituto de Ciencias de la Tierra y Ambientales de La Pampa (INCITAP), Universidad Nacional de La Pampa – Consejo Nacional de Investigaciones Científicas y Técnicas (UNLPam- CONICET), Santa Rosa, La Pampa, Argentina.
[2] Schulze Center for Novel Therapeutics, Division of Oncology Research, Mayo Clinic, Rochester, MN 55905, USA.

Table S1. a) Growth of *Acremonium* and *Sarocladium* strains with the flavonoids diosmin, herperidin and rutin as carbon source and b) Clarification halo of rutin detection.

Microorganism	a) Diosmin	Hesperidin	Rutin	b) Rutin Clarification
Acremonium sp. DMic 85104	+	+	+	+
A. curvulum DMic 01770	+	+	+	+
A. atrogriseum DMic 093497	+	+	+	+
S. strictum DMic 993190	+	+	+	+
S. kiliense DMic 00226	+	+	+	+
S. kiliense DMic 062925	-	-	-	-
S. kiliense DMic 062926	-	-	+	-
S. strictum DMic 073153	+	+	+	+
S. kiliense DMic 093549	-	+	+	-
S. strictum DMic 093557	+	+	+	+
S. kiliense DMic 103735	+	+	+	+
S. kiliense DMic 103850	+	+	+	+
S. strictum DMic 114098	+	+	+	+
S. strictum DMic 114110	+	+	+	+
S. kiliense DMic 134632	+	+	+	+
Acremonium sp. 85-2	+	+	+	-
Acremonium sp. 147-1	-	-	-	-
S. kiliense 657-1	+	+	+	+
Acremonium sp. 693-2	+	+	+	-
Acremonium sp. 780-1	+	+	+	+
S. kiliense 799-5	+	+	+	+
A. hyalinulum 862-4	+	+	+	+
Acremonium sp. 900-3	+	+	+	+
S. kiliense 937-11	-	-	+	+
S. kiliense 937-14	+	+	+	-
Acremonium sp. 959-1	+	+	+	+
Acremonium sp. 962-2	-	+	+	+
Acremonium sp. 1237-1	+	+	+	+

(+) presence (-) absence of growth or clarification halo.

Table S2. Purification of α-rhamnosyl-β-glucosidase from *Sarocladium strictum* DMic 093557.

	**Activity (U/ml)**	**Total protein (mg)**	**Specific activity (U/mg)**	**Total Activity (U)**	**Yield (%)**	**Purification Fold**
Crude extract	0.060	3.33	0.018	6.01	100	1
Supernatant	0.033	1.98	0.016	3.36	55.55	0.88
Ammonium sulphate precipitation	0.071	11.39	0.006	0.04	1.32	0.33
Size exclusion cromatography	0.007	0.1	0.07	0.15	4.08	3.88

Fig. S1 Protein sequence of the glycoside hydrolase from *Acremonium strictum* DS1bioAY4a including predicted signal peptide. The determined tryptic peptide sequence is shown after alignment.

```
MRSSTLTVLG LALSGASTAQ NTTRGHIGNV NLDAWQDAYD KAVSFVEGLS LADKARVVTG RNATSNGEFF PGLNIMDGQQ
Signal peptide
GILNSFFVTG WSQPGALTMS WDREALYAQS YAIAREFYIR GYHVVDGPTV SPGGRTAWNG RASEGFAADS YLTGVAFGES
VRAEVEAGTI SAGKHFLLYE QENNRTARAA NEYNFTSYNS LVDDKTIHET YLAPWYDGVK NGLGGAMCAM NLVNGSYSCE
Peptide
NEPLLMGLLK EEAGFPGLVV PDINGQKTVE GSANGGLDWA SDQKWTPEII EDLVTSGDVT EARLDDMAIR NIVGWYHSGL
NNGSFPTKAR DDEYRPIPKS HRELNRENGA KSIVLLKNKA SALPLKSPRV MSIFGASAGP TTAGPNKAFA IADLNPGSWS
IPEVLDGHVA TPSGAGKASL QYLVTPVQAL QDRAREAQTQ FRWILNDTFV PETVGFIPGG ANTGVRPSIP GYASFSDVCI
VFINAYGGEG HDRRTLENKA QDDLVVQVAE NCPNTVVVVN TVGVRLVDKW IENDNVTAVL YSGPLGQESG NAIADVLYGD
VNPSAKLVYT IARDESDYET RPCPTVQCEF SEGNYIDYKA FDRADTEPRF EFGFGLSYTE FEYSDIAINN VTAKAGLAKG
PPSVGGRADL WDEVASISVQ ITNAGKVQGT EIAQLYLTFP EAADQPVRQL RGFDKVVLNP GETKAATFTL HRRDMSFWDV
EAQNWRLASG SYEAFVGSSS RDFRAEGEIK VDARR
```

Figure S1
